# Understanding Grief During the First-Wave of COVID-19 in the United Kingdom—A Hypothetical Approach to Challenges and Support

**DOI:** 10.3389/fsoc.2021.607645

**Published:** 2021-03-23

**Authors:** Chao Fang, Alastair Comery

**Affiliations:** University of Bath, Bath, United Kingdom

**Keywords:** COVID-19, grief, compassionate communities, bereavement support, death, the UK

## Abstract

**Purpose:** This article develops immediate understandings of loss and grief at both an individual and collective level following the first-wave of COVID-19 in the UK. This allows for insights into the likely challenges and support for loss and grief in facing unprecedented disruption and uncertainty. Ultimately, it explores avenues for the priorities to inform better bereavement support.

**Methods:** By examining trusted media data and carefully selected academic literature, we analyse both individual and societal responses to loss and grief in the novel context of the first-wave of COVID-19 in the UK. The discussion relocates the ideas of good and bad deaths in the context of increased social constrains and inequalities. Further, two pairs of contrasting hypotheses are proposed to examine how the UK's first-wave outbreak has shaped policy and practical structures and how these have further impacted experiences of loss and grief both at an individual and collective level.

**Findings:** The discussion captures a mixed picture of loss and grief in the UK, which highlights the importance of timely, holistic, and continuous support both in social policy and care provision. It is found that individuals and collectives express diverse needs in response to deaths and losses as a process of meaning-making. Further, the significance of socio-cultural environments also become evident. These findings highlight community support during the outbreak and further promote a grief literate culture as imperative to support individual and collective needs when confronted with loss and grief.

**Conclusion:** This article provides a timely and comprehensive account of possible challenges and support both for individual and collective experiences of loss and grief at a time of unprecedented social restrictions and mass deaths in the UK. These understandings provide a base from which we advocate the priorities for future research into the ongoing impacts of COVID-19 on grief and bereavement.

## Introduction

COVID-19 has claimed over 40,000 lives in the first wave outbreak (approximately between March to June, 2020) in the United Kingdom (Office for National Statistics, 2020a). First reported in Wuhan, China in December 2019, this highly infectious new coronavirus was first confirmed in the UK in late January 2020. It soon spread across the country resulting in a sharp increase in the number of hospitalizations and deaths. This has led to an extended national lockdown, causing significant economic disruption, and social restrictions. Meanwhile, the vast number of deaths during this period have also caused tremendous disruption and distress, widely affecting the stability and consistency of individual lives, communities, and society as a whole.

Given the unprecedented challenges, uncertainties and isolation faced in the first wave COVID-19 outbreak, it is not unreasonable to speculate that death, dying and bereavement in the UK could be greatly impacted. As widely reported by media outlets, many patients in Britain and across the world have had to die alone without the company of their loved ones, at home, in hospitals, hospices, or other care facilities (e.g., Sky News, 2020a). Frustration and even desperation may be faced by the survivors who are unable to say goodbye to, care for, or pay their last respects to their loved one. Further, lockdown and social distancing rules may strongly restrict bereaved people's capacity to seek emotional closeness and social connectedness when facing the above experiences of loss and grieving. As such, the painful experiences of loss and powerlessness may trigger unbearable sorrow, regret, and even anger for bereaved people and could cause further difficulties in coping with loss in their ongoing lives (Holst-Warhaft, 2000; Valentine, 2009). Despite the extraordinary levels of social disconnection and constraints seen during the first wave, bereaved people may receive more support, both practical and emotional, from kin, friends, neighbors, and the wider society because of interpersonal bonds and social solidarity. These positive and supportive responses may be also seen at a more collective level through the heroisation of deaths and public mourning. As such, responses to losses and deaths in the context of COVID-19 in the UK are not only individual but also societal.

To begin to develop nuanced understandings of loss and grief during and post the initial outbreak, this article situates a reflexive discussion of experiences and responses to loss and grief in the context of COVID-19. The focus on the first wave is of particular importance as many unprecedented challenges were faced by people in the UK for the first time. Since this article is written throughout the first wave when primary data collection is difficult and secondary academic data is extremely limited, the authors draw upon media coverage from trusted sources to capture an immediate “real-world” picture of dying and grief experiences. By posing two sets of diametrically opposed hypotheses, this paper envisages the challenges and support of dealing with loss and at both an individual and societal level. In so doing, this article aims to critically review the provision of support for loss and grief during the first wave in the UK. Further, it identifies priorities for future research, practice, and policy-making to better support individual loss and mass death as the COVID-19 pandemic continues.

## Conceptualizing Grief in the British Context

The loss of a loved one or a significant member of a collective can be highly distressing and disruptive, often requiring rigorous readjustments (Walter, 1999). Despite the prevalent impacts of loss, people's experiences of it can be extremely diverse and have been studied from a multitude of dimensions. Grief can be understood as complex experiences of “psychological, behavioral, social, and physical reactions following the death of a loved one” (Stroebe, 2018, p. 71). Bereavement is broadly defined in Western writings as “the objective state of having lost someone or something,” which acknowledges the sociocultural impacts on bereaved people after losing a loved one in their everyday life (Walter, 1999, p. 4). Compared to notions of grief and bereavement, mourning is rather specific, highlighting rituals, and customs that are deeply rooted in socio-cultural expectations and prescriptions for responding to loss and disruption (Durkheim, 1912; van Gennep, 1960). To capture an immediate picture of experiences of loss during the first COVID-19 outbreak in the UK, we focus on the socio-emotional dynamics of *grief* , exploring how individuals, and wider society have mediated their emotional responses to loss through the available socio-cultural discourses.

Grief discourses in the UK have largely mirrored the prevalent medicalization in contemporary British society, which often emphasizes the clinic lore of pursuing “normal” grieving and “healthy” grief outcomes (Walter, 1999). This uptake in scientific and rationalist understandings of loss and grief has been furthered by the decline of religious influence and other traditional values in how we feel, understand, and interpret loss (Walter, 1996; Hockey, 2001). As such, individuals' grief in Britain has been constructed as a rationalized and regulated process where one can “complete” the experience and as such return to a “normal mental state” (Valentine, 2008). This process is often assisted by psychological coping frameworks and professional interventions such as therapy, counseling, and even medication (Walter, 2007). The medicalization is also evident in so-called “dying trajectories,” which highlight the strong involvement and sometimes domination of medicine and professionalization in the dying process in western countries (Strauss, 1971). As such, the dying person and their loved ones may find little space left for them to reaffirm bonds and negotiate meaning for the painful aspects of dying. These highly medicalised and institutionalized experiences (e.g., dying alone in hospital) are also often associated with the construction of bad death, further imposing challenges for the survivors to make sense of loss and grieve in their ongoing lives (Lawton, 2000; Seale, 2004). Despite the prevalent influences of medicalization in British people's experiences of loss and grief, studies have also highlighted the significant roles of everyday interactions and support in helping bereaved people (Walter, 1996; Valentine, 2008; Neimeyer, 2011). Central to this everyday approach is to understand how bereaved people negotiate with the available socio-cultural discourses to grieve and how everyday interactions can support this meaning-making process without necessarily relying on formalized interventions (Valentine, 2008).

Alongside medicalization, grief in the UK has also been highly individualized (Walter, 1999). Living in an increasingly diversified and individualized society where heterogenous values and norms are no longer predominant, bereaved people are more likely to confront their loss and grief by drawing upon norms from their associated sub-groups and based on their personal interests, religion, race, and so on (Giddens, 1991; Walter, 2007). Studies have found that bereaved people in British society may feel disorientated due to the lack of strong socio-cultural instructions for dealing with loss. Meanwhile, they may also actively adopt, revise, and incorporate varied resources from themselves and wider society to develop a more personalized approach to grieving, such as personally tailored rituals (Bradbury, 2001; Valentine, 2008). The individualization of grief has also paradoxically prompted the increased emphasis on social support for bereaved individuals, often through family, self-help groups, local communities, and the internet (Walter et al., 2017; Breen et al., 2020). As such, the British experience of individual grief increasingly intersects with community and social support which can provide an empathetic and compassionate approach, through which bereaved people's emotions can be understood and shared by others (Kellehear, 2005).

Shared experiences of loss and grief can also give rise to so-called collective grief, a phenomenon that can be seen when facing the death of a public figure or mass deaths in the UK as in many other countries. Often through publicly available mourning rituals or platforms, the British people have assembled to honor and remember the deceased at times when society experiences significant loss, such as the World Wars, terror attacks, and the death of Diana, Princess of Wales (Walter, 2020). These collective responses for expressing grief and pain have helped society as a whole restore stability and solidarity preventing major disruption to social orders (Davies, 1999). Meanwhile, these shared experiences also have particular meaning for British people in the highly individualized context. By symbolically interacting with others in society, collective grief can provide an emotionally mediated and socially constructed means for many British people to reaffirm their collective bonds with wider society (Alexander et al., 2004).

## Methods

By understanding grief as a socio-emotionally challenging experience facing both individuals and wider society, this article presents an immediate response that critically reviews reliable media data regarding the initial impacts of COVID-19 on loss and grief in the UK's first wave outbreak. As such, we aim to explore the experiences of death and grief during the first wave and how these experiences could be shaped by the challenges presented in light of the COVID-19 pandemic. We also analyse the diverse support for grief and grieving, capturing the duality of such experiences. The analysis is further reinforced by a hypothetical lens, actualised via the use of two pairs of diametrically opposed hypotheses, to envisage how existing academic understandings of bereavement can be situated within this novel context. The opposing nature of each pair of hypotheses situates this article to vividly capture the diverse and dynamic nature of responses to loss and grief at both an individual and societal level.

Given the immediate nature of this article, research on the impacts of the COVID-19 pandemic on loss and grief was extremely limited. As such, a rapid review of academic literature was conducted using the search parameters of peer reviewed articles between 1990 and May 2020 following the guidance of the WHO 2017 framework (Tricco et al., 2017). The search terms used were “grief,” “bereavement,” “mourning,” “mass death,” and “pandemic.” The authors consulted the resulting articles to identify pre-existing theoretical and empirical understandings that are applicable to the experiences of loss and grief during this initial outbreak in the UK.

To further illustrate our findings, we draw upon reliable media reports to explore and further understand attitudes, responses, and practice in the context of the significant numbers of COVID-19 related deaths. In order to ensure the validity of the media data, we only draw upon data from three trusted sources, namely BBC, ITV, and Sky News in alignment with academic analysis of digital news (Kousha and Thelwall, 2017; Newman et al., 2020). All media data used were cross-checked by both authors to ensure that any claims made were not disputed by the publicly available government data. All data sourced from the media was collated daily during manual searches of the three selected trusted media sources throughout the first wave.

By employing a hypothetical approach to frame challenges and support experienced by bereaved individuals and wider society, we identify potential gaps in support for loss and grief during the first wave of COVID-19 in the British context. In so doing, we further conceptualize the priority issues for the ongoing policy agendas in relation to grief and bereavement support during and post the COVID-19 outbreak in the UK, advocating for future research to explore policy agendas that best address the current weaknesses in support.

## Good and Bad COVID-19 Deaths

Exceptional to COVID-19 is the severity of virus transmission, and the unprecedented levels of social restrictions imposed during the outbreak could undermine people's autonomy and the support resources available to them in the face of death and dying. Restrictions of this nature have not been seen in other pandemics and mass death events in the UK. Furthermore, Covid-19 deaths are likely to violate predominant public and healthcare discourses about “good death,” which favor a pain-free and smooth dying process emphasizing holistic well-being, family presence, autonomy, and dignity (Meier et al., 2017). Despite “bad death” prevailing in this pandemic, “good death” may be possible as an unforeseen consequence of the increase of home death due to restrictions placed on medical and care facilities. Good deaths have also been constructed in media and public discourses as heroic and thus good, as a collective means to justify losses and alleviate emotional costs during Covid-19.

### Painful Death

Contracting COVID-19 may cause debilitating complications with symptoms including pneumonia, dyspnoea, acute cardiac injury, and other secondary infections (Huang et al., 2020). Many patients have reportedly experienced tremendous discomfort, including severe difficulties in breathing, having to rely on ventilators during their final moments (BBC News, 2020g). This suffering could be further exacerbated during the early stages of the first wave when medical resources were extremely limited. In early April 2020, the British Medical Association advised doctors to prioritize ventilators and other care resources to focus on those with the best chance of survival (ITV News, 2020a). As such, this approach could potentially leave older people and fragile patients with little room to seek relief, thus likely dying with extensive physical deterioration, discomfort, and distress. Pain of this nature could also be experienced by non COVID-19 cancer and terminal patients whose life-saving/sustaining care was delayed or canceled due to the disruptions caused by the outbreak (BBC News, 2020h).

### Lonely Death

The painful nature of deaths during the first wave outbreak in the UK could be further escalated by forced separation from loved ones resulting from the strict prevention and control measures. As such, dying patients could face increased risk of suffering lonely death, an experience of not only dying physically alone but also in a marginalized status of being socially isolated and emotionally lonely (Turner and Caswell, 2020). When experiencing physical deterioration and having lost the capacity to care for themselves, dying patients could be forcedly barred from seeking and gaining support and care from their loved ones due to COVID-19 quarantine measures. Family support at the deathbed can often forge cherished moments that enable the dying person and their loved ones to gain comfort and to reaffirm their family bonds (Lawton, 2000). These experiences may allow for a meaningful passage to transform and heighten their bonds not only through religious or spiritual norms but also simply by physical contact and language, such as a kiss or the words “I love you” (Pace and Mobley, 2016). These symbolic and intimate interactions may be difficult, even impossible under the contagion control measures evident during the first national lockdown. Although many care providers relied on video calls to help families say farewell to their loved one during the outbreak, these interactions could be greatly limited. A bereaved son from the UK conveyed his frustration with the virtual farewell to his mother (ITV News, 2020c):

*I wanted to go there but I wasn't allowed and that's the hardest thing, just not to be able to comfort her and stroke her head and kiss her and just to be able to hold her hand*.

Dying with extensive physical pain and emotional distress and also with little support can be extremely undignified (Seale, 2004). Although restrictions were relaxed in the UK post the peak of the first wave allowing family members some access to see their dying relatives in care facilities (BBC News, 2020b), by this point many people had already died unaccompanied by their family.

The risk of experiencing a lonely death could be further amplified for those who had been vulnerable and disadvantaged before the outbreak. People from low-income backgrounds and ethnic minority groups have also been reportedly the worst hit communities by COVID-19 related deaths in the UK (e.g., Bear et al., 2020; Public Health England, 2020). Social inequalities have greatly contributed to the high death rates among those isolated and disadvantaged. Without sufficient policy consideration and social support, these deaths could be both lonely and also marginalized, questioning social responses, and broader structures in the context of the pandemic (Kellehear, 2007).

### Unexpected Death

During the first wave of COVID-19 in the UK, many people have died unexpectedly within weeks and even days after contracting the virus. In the face of sudden deaths, bereaved people are often left little time to face and prepare for their loss. Research has found that the tragic and unexpected loss of a loved one could place survivors at increased risk of experiencing a sense of incomprehension, helplessness, and guilt (Holst-Warhaft, 2000; Valentine, 2010). This loss may also acutely question bereaved people's taken-for-granted life, family, and social relationships, further challenging their sense of meaning and identity (Handsley, 2001). The unexpectedness of loss could be even more pronounced in the face of premature deaths. Media and public discourses have strongly portrayed COVID-19 as a serious threat to the lives of the elderly and fragile, despite deaths of children, and those from younger generations being recorded. Child deaths are often considered untimely, unnatural therefore particularly bad, likely leaving the bereaved family and broader society struggling to justify the loss (Walter, 1999). The pain and isolation attributed to COVID-19 could further intensify the debilitating nature of child loss, as conveyed in an interview about a teenage victim who died suddenly after contracting the virus in London (ITV News, 2020b):

He was a young boy, 13 without his mother, without any of his siblings on his deathbed in the last moments. That's very hard to understand and digest…

### Home Death

Whilst COVID-19 and its resulting pain and restrictions have given rise to experiences of “bad death” for many in the UK during the first wave, the outbreak has also paradoxically allowed opportunities for some people (e.g., terminally ill patients) to die at home. The place of death has long been used as a key indicator for the quality of end of life care in western countries, suggesting home as an ideal environment for the dying person to receive emotional comfort and dignity (Seale, 2004; Meier et al., 2017). As reported by the Office for National Statistics (2020b), deaths in private home increased 40.3% between 1st March and 30 April 2020, compared to the average of previous the 5 years. This increase in home deaths may have been caused by hospital disruptions and patients' fears for contracting COVID-19. This decision to remain at home could however enable them to spend their last time in a familiar environment and accompanied by family (from the same household/social bubble). Despite the prospect of experiencing “good” home death, this could only be possible if the patient can receive sufficient palliative care in this environment. Given the delays and cancellation of care faced by many home-bound patients during the first outbreak (BBC News, 2020j), it remains unclear to what extent home deaths were “good” in the context of the first-wave outbreak.

### Heroic Death

The good nature of death may also be possible at a more collective level. When facing mass deaths and crises confronted in this pandemic and other catastrophic events in the UK, such as disasters, wars, terror attacks, and pandemics, it could be particularly important to provide meaningful scripts to both support individual losses and reinforce social solidarity (Seale, 1995; Walter, 2020). Heroic deaths have been constructed and promoted by media and public discourses during the outbreak, to honor healthcare and other essential workers (Atlani-Duault et al., 2020). For example, the sacrifices of many other key contributors, who have lost their lives to save and help others, have also been honored in a memorial list in the UK (BBC News, 2020i). The recognition of their professional identity and selfless spirit can offer meaningful reminders of hope and wholeness to society as an entity (Goren, 2007; BBC News, 2020c; Walter, 2020).

## Challenges and Support in Facing Grief and Bereavement

Significant challenges and risks when dealing with death and grief could be expected at both an individual and societal level both during and post the first-wave COVID-19 outbreak in the UK. As outlined above, deaths during this outbreak could often be “bad,” violating the modern craft of dying in the UK which emphasizes individual autonomy, holistic support, preparedness, and physical togetherness with family (Walter, 2020). Due to lockdown, social distancing, and other new restrictions, bereaved people may find their needs for grieving and bereavement largely unattended. The increased social inequalities seen during this outbreak may contribute to or even exacerbate barriers for grieving and bereavement. These difficulties could be particularly evident for vulnerable and disadvantaged individuals and is likely to restrict their access to external support (Bear et al., 2020). In contrast, support at a community level may become more available and vibrant during COVID-19 (Office for National Statistics, 2020c). Grassroots support and mutual understandings were seen in local settings, while public mourning has emerged across the UK. Although this increased support may help bereaved people confront and deal with their grief and bereavement, it is unclear to what extent and in what circumstances this support could impact bereavement experiences. Little academic data has been generated during the first wave to gain explicit understandings. To develop the broadest possible picture of COVID-19 related grief in this immediate response to the first wave, we employ two pairs of diametrically opposed hypotheses to evaluate possible challenges and support for grief and bereavement both at an individual and collective level.

## Individual Grief

Previous pandemics that significantly impacted the UK, such as HIV, not only caused wide spread deaths and fear but also created invisible barriers for survivors to exercise their individual agency to cope with their loss (Sherr et al., 1992; Bristowe et al., 2016)^1^. Similarly, the COVID-19 pandemic could amplify the conflict between individual needs and public health interests due to adverse social restrictions and inadequate support. Some bereaved people may feel more powerless and isolated and as such grieving for their loved one could be difficult or even impossible. However, informal support from kin, friends, neighbors, and colleagues may remain available or become more accessible at this difficult time. Given the large death toll within the UK, victims' families, and friends may also form and share symbolic bonds, as seen in bereaved families of deceased veterans (Walter, 2020). To further explore the possible impacts of COVID-19 on individual grief, two contrasting hypotheses are proposed to examine relevant media coverage and research: COVID-19 can make grieving harder or easier compared to experiences of grief prior to this pandemic.

### Hypothesis 1: Grieving and Bereavement Are Harder in the Context of COVID-19

Bereaved people in the first wave may face isolation and inability due to national lockdown and other control measures. As mentioned above, bereaved people could struggle to find meaning while confronting a bad death of their loved one which may be painful, lonely, unexpected, or a combination of these. The increased social restrictions seen during this period could further compound the difficulties in bereaved people's experiences of meaning-making. Thus, grieving for their loved one could be difficult or even impossible. This poses two questions, does this sense of powerlessness and helplessness in grieving persist even after the death? Does it obstruct bereaved people from seeking comfort from others and developing mutual support networks to cope with grief? The following quote from an interview with a bereaved son may allude to one possible answer (ITV News, 2020c):

*We haven't been able to see each other, we haven't been able to comfort each other, or grieve properly. We are all isolating. We can't go out, can't comfort each other*.

In response to the tight control measures, bereaved people may experience limited agency to deal with their grief following the death of their loved one. Funerals and ceremonies have been canceled, postponed, or significantly altered (e.g., BBC News, 2020o). For example, funerary rites may have to be minimalised or held online. These restricted and virtual interplays could hardly replace normal face-to-face interactions and physical memorial activities, which involve not only close family members but also those from broader social networks. A hug, a conversation with other mourners or a sacred site of religion may allow for and even heighten special emotions and meanings for bereaved people (Walter, 1996; Davies, 2017). Therefore, bereaved people may find the absence or minimality of funerary rites distressing (O'Rourke et al., 2011). What may be more distressing is the absence of an ongoing structure for mourning, such as, not being able to say goodbye to their loved one, agonizing over being separated from others at the funeral and not being able to visit the grave afterwards. Such compounded experiences of powerlessness could further have lasting impacts on their experiences of making sense of loss.

Public recognition of some people's loss and grief may also be absent during the outbreak, as not everyone's loss and grief would be automatically recognized. As such, feeling unentitled and unsupported to publicly share and cope with grief may cause disenfranchisement (Doka, 1989). Healthcare workers, as a result of their increased chances of encountering deaths in the workplace, may experience grief in relation to deaths of individual patients, colleagues, or the loss of life in a more collective sense. As such, they could be at heightened risk of experiencing disfranchisement of grief. Their professional identities may create invisible barriers making them fail to acknowledge and cope with their grief (Aloi, 2011). This may be further exacerbated by the lack of language for grief and concerns regarding professional boundaries (Lathrop, 2017). Their disfranchisement could be further amplified by health workers' agonizing life and death decisions, prioritizing care for limited patients due to restricted medical resources. A doctor conveyed his stress to BBC News (2020e): “*Seeing people die is not the issue. We're trained to deal with death… The issue is giving up on people we wouldn't normally give up on.”* Feelings of guilt, powerlessness and shame may be so strong that individuals' personal psyche, moral values, and professional identities could be severely challenged (Dean et al., 2019). Another high-risk group for disfranchised grief is bereaved family members whose loss and grief are infrequently recognized in public discourses during the outbreak. For example, a bereaved daughter felt forgotten and heartbroken when facing her mother's death only as a “figure” in official data (BBC News, 2020d):

*She was one of the figures of death. And it's heart-breaking because to everyone else, that's just a number but that number was my mum*.

Some bereaved families could experience more hardship when their loved one's death is not officially recorded in the data of COVID-19 victims (Sky News, 2020b). They may feel they are unentitled to access sympathy, condolence and other resources for COVID-19 victims and as a result, they may experience marginalization in facing their grief.

Marginalization could further exacerbate the difficulties of bereaved people facing disadvantaged deaths. As mentioned above, disadvantaged deaths may grow in black, ethnic minority and older groups as a result of increased inequalities, such as racism and social neglect during COVID-19 (Bear et al., 2020; Public Health England, 2020). According to the Office for National Statistics (2020d), “mortality rates are normally higher in more deprived areas, but COVID-19 appears to be increasing this effect.” As a direct result of this disparity in mortality rates, “more than 70 public figures are calling for a full independent public inquiry into deaths from COVID-19 among people from ethnic minority backgrounds” (BBC News, 2020l). This call for a public enquiry aims to help grief-stricken families of these victims who may have to face difficulties in understanding and justifying the death of their loved one. Inept communication and cooperation between care providers and public health authorities may also contribute to disadvantaged deaths, thus bereaved families may further question broader social structures. For example, a growing number of bereaved individuals are calling for a public enquiry into the failures of the response to COVID-19 related deaths in the UK (BBC News, 2020m). How to justify these disadvantaged deaths may remain a challenge for the bereaved in the longer term, requiring further negotiations to contest the meanings of their loss (Holst-Warhaft, 2000).

There may also be marginalization of bereavement in the context of social inequalities, leaving many bereaved people little access to support. One pandemic is essentially many epidemics; thus, challenges and available resources for grief and bereavement during COVID-19 could vary drastically between regions and even across communities, many of which have developed multifaceted responses to offer diverse support for bereaved people. These resources could both continue and further develop during and post the outbreak, including counseling services, psychotherapy, charity support, social policies, and other socio-cultural norms (i.e., Bristih Psychological Association, 2020; Cruse Breavement Care, 2020). Conversely, bereaved people in different regions and communities with limited resources and infrastructures may struggle to comprehend their loss and access relevant support (Fang, 2019).

In addition to increased social restrictions and inequalities, particular types of loss could make grieving and bereavement harder. Child deaths as a result of COVID-19 could be extremely difficult for parents, who may find their grief particularly challenging in the face of unnatural, untimely and bad death. Living in a society where premature deaths are no longer predominant, bereaved families may find limited support from social norms and religious values, to make sense of and give meaning to their child's death (Walter, 1999). The COVID-19 outbreak may further suppress their agency and resourcefulness when dealing with grief. Child deaths could also fundamentally challenge parents' identity and further destabilize family structures (Riches and Dawson, 2000; Fang, 2020). Furthermore, given the highly infectious nature of COVID-19, some people may lose multiple individuals in their family and immediate social circles. Therefore, they may experience even more compounded emotional distresses and other practical or financial issues.

### Hypothesis 2: Grieving and Bereavement Are Easier in the Context of COVID-19

Despite numerous issues and risks faced by the bereaved, people experiencing grief and loss may receive more support at this difficult time. There has been significant evidence of increased support in families, neighborhoods, and wider social and public spheres during the first wave in the UK. This raises a question: does this increased support make grief and bereavement easier compared to the pre-COVID-19 time? The answer to this question is rather complicated in light of bereavement as a diverse experience embedded in one's ongoing life. Central to understanding the diverse experiences of bereaved people in the outbreak is to examine the quality and continuity of support both in the short and longer term.

Some people's family resources and social capital could grow in the face of loss and forced separation during COVID-19. As widely seen, family and friends have provided emotional and social support via video calls and social media. Neighborhoods have also come together to offer practical and emotional support for isolated and vulnerable residents (Office for National Statistics, 2020c). Even when their individual agency was challenged by the lockdown and social distancing rules, some people exploited limited resources to access family bonds and community spirit to cope with loss. Funerals, for example, have had to be minimalised during the outbreak, but some families could have experienced a heightened sense of intimacy and solidarity through smaller family-centered funerals. For some, alternative funeral arrangements such as socially distanced memorials from home were a way to come together to reaffirm family ties in spite of restrictions. For instance, a group of siblings held a “funeral” for their deceased brother in their mother's garden, which “put a smile on my [their] mum's face at such a sad time for her” (BBC News, 2020n). Such intimate experiences may have enabled kin to better support each other to face and make sense of their loss.

In addition, condolence paid to COVID-19 victims from the wider society may have provided bereaved people a sense of sympathy and comfort. The identification with other mourners of COVID-19 victims could also help them enfranchise their grief, further contributing to developing mutual understandings and a sense of belonging in the face of meaninglessness and isolation. For example, a UK family invited other bereaved families of COVID-19 victims to use a yellow heart to visibly signal their loss and share stories of their loved one (BBC News, 2020f). In public domains, governmental bodies and professional organizations also adopted sympathetic and flexible approaches to extend their support for bereaved people. This informative and instructive support could help guide bereaved people to deal with the multifaceted issues associated with loss and grief, including financial difficulties, death registration, emotional stress and other challenges and risks. Nonetheless, not every bereaved person can access increased support and experience positive responses. Their grief and bereavement may reflect their personal circumstances and broader social contexts.

The immediate support seen during the outbreak suggests the importance and the potential of continuous care for bereaved people in the longer-term. After social distancing rules are lifted and typical social order is resumed, informal support from family, friends, colleagues, and neighbors may become more physically accessible for bereaved people. Formalized social and health care systems may continue and even expand their services for supporting bereaved people, especially in the British context where health and social care frameworks are well-established. In the longer-term, it is important to ensure professional care of psychotherapy and clinical interventions remain available where needed, to help bereaved people cope with their loss and grief. It is, however, equally crucial to avoid over-emphasizing pathological aspects of grief and heavy reliance on therapeutic frameworks (Walter, 1999; Valentine, 2008). That said, bereaved people are inherently resilient in responses to loss, tending to adopt and revise the status quo by seeking their own means of grieving (Bonanno, 2004; Valentine, 2009; Fang, 2019). Even in extremely difficult circumstances, they may still be able to transform their grief to challenge and reshape social structures (Holst-Warhaft, 2000). Bereaved people may draw upon various socially accessible tools, such as language, arts and other creative means, to reconstruct meaning as part of their ongoing lives (Walter, 1996; Neimeyer, 2011; DeNora, 2012). Meanwhile, the shared experience of loss may also prompt self-help groups both online and face-to-face in the longer term. This reciprocal support could enable bereaved people with similar experiences during COVID-19 to develop mutual understandings and a sense of belonging, thus helping them better make sense of their loss and rebuild meaningfulness both individually and collectively (Valentine, 2017; Fang, 2019). Such experiences of self-help groups may have specific pertinence for those who may not be able or may be unwilling to access more formalized support.

### Discussion: Mixed Experiences of Individual Grief and Bereavement

It is not straightforward to say if the COVID-19 pandemic has made grieving and bereavement harder or easier than before the outbreak. The answer is rather conditional, showing the complexity and fluctuation of such experiences. The COVID-19 outbreak and resultant quarantine enforcements imposed increased social restrictions. These could consequently create new barriers, suppressing bereaved people's autonomy, and restricting the available resources to deal with and recognize loss and grief. Existing issues of inequalities may also be amplified during the pandemic, adding further difficulties for some to justify and adapt to their loss. These new and pre-existing social issues could further debilitate people in the face of particularly “bad” deaths, making their grieving and bereavement even harder. Meanwhile, this difficult time has also seen increased support for bereaved people from both informal and formal sources. The current increase in short-term support as a result of COVID-19 should continue longer-term, highlighting the importance of ensuring available support for bereaved people in ongoing life.

Despite the prospect of having increased and continuing support for bereaved people, the quantity and quality of support could vary across different settings. As well as individual differences, such as individual personality and interpersonal relationships, varied environments could play a significant role in shaping bereaved people's support networks and resources (Valentine, 2008). Regional differences and inequalities between races, classes and age groups could lead to diverse and complicated experiences of grief and bereavement (Bear et al., 2020). Therefore, it is impossible to find a universal answer to the above two hypotheses. Central to this dilemma, however, is the importance of developing a more sympathetic, inclusive, and interdependent environment with grief literacy (Breen et al., 2020). This approach emphasizes a complex of resources and mechanisms enabling public and professionals to be more knowledgeable and proactively supportive in identifying and dealing with loss and grief. The above discussion, and the scenarios explored in each hypothesis, serve to inform our advocacy for more continuous and integrated support, highlighting areas where future policy and practice research attention should focus. This grief literate environment could empower bereaved people, with different needs and from various backgrounds, to better facilitate grieving and make sense of their loss in community-based and day-to-day settings. The roles of policymakers, care providers, community leaders and social activists would all be indispensable for creating this grief literate culture in communities and wider society both during and post the outbreak (Kellehear, 2005; Breen et al., 2020).

## Collective Grief

Responses to lives lost during COVID-19 may not only be individual but also collective in nature. The shared experience of loss and grief may contribute to the growing collective entities consisting of many thousands of mourners locally, nationally, and even globally. Similar experiences of loss may enable mourners to form symbolic bonds with others, thus enabling grieving in a more collective sense. This sense of belonging could also be experienced in wider society, for both the bereaved and non-bereaved, as a collective response to loss in the face of COVID-19. This collective approach to loss and grief has been widely observed across the UK and globally, ranging from the aforementioned small-scale “yellow heart” movement to public mourning for deceased healthcare workers. To better understand the phenomenon of collective grief in the UK, it is important to ask whether this has to be created through purposive strategies of idealization of deaths and communal rituals directed by the government/other public organizations, or, whether these collective responses happen as intrinsically motivated actions shared by individuals and communities? These questions could further elucidate what is needed to deal with mass deaths and shared traumas in ongoing society.

### Hypothesis 3: Collective Grieving Is a Strategic Response Prescribed by Society

Collective responses to loss could be seen as a grieving process, assembling social members to face destruction and impairment and to reaffirm their conformity to society as an orderly and functional state. Society as an entity tends to secure its stability and continuity in the face of mass deaths and social disorder. Public rituals and memorial activities can be prescribed by the government and the public to serve as transitional and functional passages intentionally helping reaffirm shared values and social solidarity among social actors (Hockey et al., 2001; Doss, 2008; Walter, 2020). One example is national mourning for COVID-19 victims held in China on 4th April 2020. This state-led grieving response provided an opportunity to remember those lost and to reunite the country, thus helping it to move forward after the national crisis (BBC News, 2020a). Although national mourning of this kind has not yet been seen in the UK on the same scale as China, it is envisaged that acts of national mourning may surface. This would resonate with the memorialization of other mass-death events, such as Remembrance Day on the 11th November each year, providing collective and symbolic means of recharging the energy and confidence of society.

Such publicly organized commemoration activities are not necessarily temporary but may persist, shaping the continuing existence of society. These events can be purposively repeated in relation to special tempo-spatial elements (Holst-Warhaft, 2000), such as the annual Remembrance Day for victims who died in the wars, organized by the governments and communities across the country. Similarly, significant locations and dates may be hallowed after the COVID-19 pandemic, to collectively, and continuously reconstruct the past and to reinforce a communal sense of belonging for the future of society (Alexander et al., 2004). For example, New York City announced the use of a long-marginalized site, Hart Island, to bury COVID-19 virus victims (BBC News, 2020p). This burial ground may later be promoted as special location because of the associated memories of mass death and crises. In the UK, the arranged annual memorial events on the site of Grenfell Tower in London, where a fire killed 72 people in 2017, continues to play an important role in the ongoing memorialization of those who lost their lives. Similarly, sites, dates and even persons with specific pertinence regarding COVID-19 may also be chosen across the country, to memorialize the mass deaths and trauma. Therefore, how to manage and transform these sites may play a significant role in reshaping the emotions of individual bereaved people and the public, allowing them to further negotiate and contest meanings both personally and collectively. Such publicly visible signs of this pandemic may appear elsewhere, presenting opportunities and challenges for both governments and communities, namely, how to transform collective pain and public emotions into meaningful shared memories of social solidarity and collective confidence.

### Hypothesis 4: Collective Grieving Is a Spontaneous Response Shared by Individuals

An alternative approach to public anxiety and tension lies in voluntary individual actions. Given that the pandemic may challenge shared values and beliefs, individuals could spontaneously seek “creative and highly idiosyncratic” ways to grieve their loss and ease their tension (Bradbury, 2001:0.221). That is, those affected by loss and grief can come together to exercise their agency to reshape the form and meaning of grief in wider society, without instructions, or pressure from external forces. These individual reactions may coincide and resonate with each other and thus naturally evolve into shared grassroots grieving activities, such as the yellow-heart movement in the UK. In addition, social media and dedicated memorial websites could provide alternative virtual platforms to create shared spaces to collectively acknowledge loss and grief during and post the outbreak. These online platforms could empower both the bereaved and non-bereaved to publicly legitimize and collectively alleviate their tension and grief (Harju, 2015). Ultimately, this could empower the public to create an online archive of reflective and meaningful lessons learnt from death and dying to reflect more broadly the resilience shown during this unprecedented pandemic.

### Discussion: Need for Collective Responses to Loss

Collective grieving activities may be both extrinsically and intrinsically motivated during this pandemic. Despite this difference, needs for such shared responses to deaths and losses are clearly revealed. Responding to collective grief entails a duality of social functions and individual autonomy, underlining the socially mediated processes to deal with loss and grief at a societal level (Valentine, 2008; Fang, 2019). From a structural perspective, society, especially the authorities, may prescribe norms to allow for and regulate publicly acceptable emotions through public commemorations. These could serve to recover social order and solidarity following aggregate losses during COVID-19. In light of individual agency, social members may coincide or deliberately plan to seek and create platforms to express their sorrow for collective loss and heroic individual deaths, further restoring their social identity. Both externally organized and spontaneous responses to collective grief have been documented during the outbreak and may continue to occur post the outbreak in the UK and elsewhere. Collective grieving may sometimes become difficult and even impossible due to lacking the appropriate resources and structures to support these actions. For example, some local authorities may not be financially able to support public memorial events, while some individuals may feel difficulty in expressing their grief in public due to little emotional support in their community. As such, it is essential to ensure available channels for both society as a whole and its members to restore meaning and an equilibrium in a collective and symbolic sense. The importance of social environments is again evident, calling for a resourceful, supportive and grief literate culture (Breen et al., 2020). An environment of this nature could enfranchise society and individuals to negotiate meaningful ways to respond to the evident need for collective grief. Meanwhile, the shared experience of COVID-19 may further reinforce individual bereavement experiences by providing more established collective norms and values to reaffirm their social membership and enfranchise feelings of loss and grief.

## Concluding Remarks and Avenues for Future Research

The primary aim of this article is to adopt a hypothetical approach to explore possible challenges and support experienced by those affected by loss during the first wave of COVID-19 in the UK. By examining trusted media data and carefully selected academic literature, the discussion revisits and further relocates the ideas of good and bad deaths, grieving and bereavement support. These are examined in the context of the changing dynamics of social discourses and individual experiences as a result of the first wave. Both good and bad deaths are explained and analyzed to provide foundational understandings for the challenges and opportunities involved in deaths in this novel context. A largely “bad” nature of deaths is captured, often involving pain, loneliness, isolation and unexpectedness. “Goodness” is also seen through the increased potential of being able to die at home and heroisation of deaths, showing the diverse, and even competing experiences of death and grief that people may confront.

Due to lockdown, social distancing and other new norms developed during the first wave in the UK, pre-existing structures of mourning and grieving may become largely absent. This may require revised, compromised or completely new ways to grieve and to deal with bereavement. Despite lacking primary empirical data, it is still possible to develop a preliminary and exploratory view of loss and grief in this context. By carefully drawing upon media and academic discourses, a mixed picture of both negative and positive experiences of grief are captured at both individual and collective levels. To better understand the complexity, two pairs of contrasting hypotheses are proposed to examine the impacts of COVID-19 on experiences of loss and support. As discussed, bereaved individuals may face both improved support or intensified challenges depending on their individual circumstances and social backgrounds. The social environment for grief and bereavement is found to be particularly important. This is also evident in experiences of collective grief, in which relationships between broad structures and individual agency could powerfully shape the means of shared responses to deaths and losses.

Despite the varied nature of grief reactions captured in the two pairs of hypotheses, two primary findings have become evident. These are: (1) the diverse needs of both individuals and collectives to cope with and make sense of loss and grief, (2) the significance of socio-cultural environment in the process of coping with grief. Furthermore, the question of how to better respond to these varied needs for grief is fundamentally entwined within the social environment in which it is situated. To better support those experiencing loss and grief in an ongoing manner, grief literacy may play a pivotal role in allowing for mutual understandings within the community to better support the everyday lives of those affected by loss (Breen et al., 2020). This idea forms part of a broader framework of compassionate communities and is motivated by the increasing professionalization and inequalities in bereavement care (Kellehear, 2005). A multifaceted approach would be required to foster grief-literate environments across the UK both during and post the outbreak.

The increased awareness of our own mortality as a result of the mass death seen during the COVID-19 pandemic may shape the way in which we view grief and bereavement moving forward. Increased media coverage of the loss of life and bereavement in the UK may contribute to developing the understanding of grief and appropriate support tools, including language, music and other creative means, to help deal with loss. This may further help facilitate a more compassionate and grief literate society (Breen et al., 2020). It is important to note that despite the current restrictions, specialist support, and guidance regarding grief and bereavement remain available from charities, government organizations, and care providers. However, the significant role played by local communities should not be underestimated as they have continued to help develop appropriate structures and integrate relevant resources for those experiencing loss, in their immediate social networks. Further, situating experiences of loss and grief within their localized contexts can allow for more inclusive and individualized approaches to reinforce community-based support. As such, these embedded approaches would direct resources to more adequately tailor support to respond to the diverse needs for grief and bereavement at both an individual and collective level, better accounting for different social, religious, ethnic, age, and gender groups.

The self-help group model could provide an invaluable framework to highlight the importance of localized support in non-psychotherapeutic settings, uniting people with similar backgrounds and mutual understandings in their bereavement experiences. This framework could also empower people to deal with their loss in a more spontaneous and proactive manner in everyday life, further encouraging grief literacy in wider society. Such integrated and localized approaches could help supplement the support and guidance provided by the government and professional social and health care. Meanwhile, these more formalized support frameworks could also complement and reinforce community-based support. Cooperation between these two avenues could allow for a “new” structure of bereavement support that would more comprehensively address grief and bereavement in light of the significant ongoing challenges posed by COVID-19 in the UK.

Based on the findings of our hypothetical approach, we indicate an agenda for future research that may guide and shape future practice and policy-making for the ongoing battles with COVID-19 and other similar crises. To resonate with the above hypotheses, we suggest that two key questions can be asked in future inquires: (a) what is needed in response to loss and grief at both an individual and collective level when facing significant social restrictions and mass deaths, (b) how do social environments support or undermine these needs in this novel context. Future research should aim to collect empirical data, which was not possible in this immediate response. More specifically, it should employ both quantitative and qualitative approaches to clarify the enablers and barriers in individual and collective grief experiences. These enablers and barriers should also be examined within the context of minority and disadvantaged groups within the broader population. Further investigations on bereavement for particular demographic groups, such as children, adolescents and older people, are both important and necessary to understand the impacts of COVID-19 on the social constructions of loss and grief. Comprehensive research of this nature would further ensure that future support provision is more appropriately tailored to its end-users and is tied to its broader cultural context.

In addition, studies on collective responses to deaths as a result of COVID-19 could offer powerful and creative insights into the ongoing debates on public mourning and memorialization in the UK. The ideas of grief literate societies and compassionate communities are worth further consideration, to explore how community-based support could complement pre-established statutory support during pandemics and other major emergency events. Meanwhile, the importance of language and other socially accessible tools, such as the arts, should also be considered in future policy-making and care provision discussions moving forward. This approach may provide more accessible resources for bereaved people to deal with their loss and grief during and post the outbreak. This article provides a base from which we advocate the priorities for future research into the ongoing impacts of COVID-19 on grief and bereavement on both an individual and collective level. Although this article focuses on the British context, some lessons about the diverse grief needs and compassionate social environments may also be relevant in conducting similar research in other socio-cultural contexts.

## Author Contributions

CF has taken the responsibility to develop the overall structure of this article, as well as to write the section individual grief. AC has contributed to the section collective grief. Both authors have worked closely to collect data, develop analytical discussions, write up this manuscript, and have jointly written up the remaining sections of this manuscrpit.

## Conflict of Interest

The authors declare that the research was conducted in the absence of any commercial or financial relationships that could be construed as a potential conflict of interest.

## Footnotes

^1^Deaths in previous pandemics, such as HIV, could be associated with stigma (Bigelow and Hollinger, 1996). While COVID-19 related deaths may be stigmatized in other countries, we have not found this to be a major issue in the UK. However, some racial stigma has been recorded aimed toward Chinese and Asian people in the UK (BBC News, 2020k).
